# Prediction of the Soil Organic Matter (SOM) Content from Moist Soil Using Synchronous Two-Dimensional Correlation Spectroscopy (2D-COS) Analysis

**DOI:** 10.3390/s20174822

**Published:** 2020-08-26

**Authors:** Shifang Wang, Xu Cheng, Decong Zheng, Haiyan Song, Ping Han, Peter Yuen

**Affiliations:** 1 College of Agricultural Engineering, Shanxi Agricultural University, Taigu 030801, China; wangsf@brcast.org.cn (S.W.); lanzhou2009chunjie@163.com (X.C.); zhengdecong@sxau.edu.cn (D.Z.); 2 Beijing Research Center for Agriculture Standards and Testing, Beijing Academy of Agriculture and Forestry Science, Beijing 100097, China; hanp@brcast.org.cn; 3 Beijing Municipal Key Laboratory of Agricultural Environment Monitoring, Beijing 100097, China; 4 Agricultural Mechanization Schools in Shanxi Province, Pingyao 031100, China; 5 College of Software Engineering, Shanxi Agricultural University, Taigu 030801, China; p.yuen@cranfield.ac.uk; 6 Centre of Electronic Warfare, Cranfield University, Shrivenham, Swindon SN6 2LA, UK

**Keywords:** two-dimensional correlation spectroscopy, visible-near infrared spectroscopy, partial least square regression, spectral variable selection, moisture effect, soil, soil organic matter

## Abstract

This paper illustrates a simple yet effective spectroscopic technique for the prediction of soil organic matter (SOM) from moist soil through the synchronous 2D correlation spectroscopy (2D-COS) analysis. In the moist soil system, the strong overlap between the water absorption peaks and the SOM characteristic features in the visible-near infrared (Vis-NIR) spectral region have long been recognised as one of the main factors that causes significant errors in the prediction of the SOM content. The aim of the paper is to illustrate how the tangling effects due to the moisture and the SOM can be unveiled under 2D-COS through a sequential correlogram analysis of the two perturbation variables (i.e., the moisture and the SOM) independently. The main outcome from the 2D-COS analysis is the discovery of SOM-related bands at the 597 nm, 1646 nm and 2138 nm, together with the predominant water absorbance feature at the 1934 nm and the relatively less important ones at 1447 nm and 2210 nm. This information is then utilised to build partial least square regression (PLSR) models for the prediction of the SOM content. The experiment has shown that by discarding noisy bands adjacent to the SOM features, and the removal of the water absorption bands, the determination coefficient of prediction (*R*_p_^2^) and the ratio of prediction to deviation (RPD) for the prediction of SOM from moist soil have achieved *R*_p_^2^ = 0.92 and the RPD = 3.19, both of which are about 5% better than that of using all bands for building the PLSR model. The very high RPD (=3.19) obtained in this study may suggest that the 2D-COS technique is effective for the analysis of complex system like the prediction of SOM from moist soil.

## 1. Introduction

Earth soil consists of complex mixture of substances, including organic carbon (OC), organic matter (OM), minerals, water and air. In the perspective of plant growth, the essence of the soil is the soil organic matter (SOM) and its content within a unit volume of soil quantifies its fertility for the growth of crops. SOM is mostly the crop residues added to the soil after their decompositions by soil macro-fauna and micro-organisms. As such the SOM consists of carbon, hydrogen, oxygen [1]. Thus, the technology for the rapid detection of SOM content in soil has been a hot topic in the soil research community within the past couple decades [2,3,4,5,6,7,8,9,10,11,12,13,14]. Visible-near infrared (Vis-NIR) spectroscopy has been a simple and efficient analytical technique which has been widely deployed for the detection of SOM content [15,16,17,18,19,20,21,22,23]. The high sensitivity of vis-NIR spectroscopy has been shown to be capable to detect trace minerals such as arsenic contamination in soil [24]. However, because of the strong moisture absorption peaks in the vicinity of the SOM features within the vis-NIR spectral range, the presence of moisture in the soil interferes the interpretation of SOM content significantly [25,26,27,28,29,30,31,32]. It has been reported that, the two strong moisture absorption peaks that are located at about 1470 nm and 1900 nm, and the relatively weaker water absorptions features at 600 nm, 738 nm and 836 nm [33,34,35], have induced spectral distortions of the SOM absorption features particularly in the vis-NIR spectral region at about 1400 nm and 1900 nm [25,36,37,38,39]. The distortion of the SOM spectral features induced significant errors in the assessment of the SOM content from the moist soil using spectroscopy methods [27,28,29,30,31,32,40].

There are many approaches and methodologies reported in the literature to minimise the effects due to moisture in the soil for improving the assessment accuracy of SOM via spectroscopy techniques. One approach adopts the model transfer methodology that utilises data of standard samples, such as the external parameter orthogonalization method (EPO) [25] which projects the soil spectra orthogonal to that of the moisture spectrum thus removing the moisture contribution from the data effectively. Other method utilises the direct standardisation (DS) [38,41,42,43] methodology which removes environmental factors (e.g., temperature, texture of soil surface, etc) from the spectroscopic database to make the library data more suitable for field data usage. Similar techniques such as the piecewise direct standardisation (PDS) [42,43,44,45,46,47,48,49], the slope/bias (S/B) correction [50] and the orthogonal signal correction (OSC) [50] have also been applied and better prediction of SOM have been achieved when it is compared directly with that using the raw spectral data for the analysis. Other stream of model transfer approach that utilises non-standard samples, such as the standardisation of spectra through finite impulse response (FIR) method [51], has been an alternative way to achieve similar result as that of the PDS technique. Recent research [26] has reported that the EPO, DS and the global moisture modelling (GMM) accounts for the effect of moisture in soil spectra better than that of the S/B and selective wavelength modelling (SWM) methods. All these methods, such as the EPO have been shown to improve the prediction of soil organic carbon (SOC) substantially with an increase of the coefficient of determination (*R*^2^) from almost zero when no moisture correction is made, to over 0.50 after the correction is applied [26]. Amongst these studies [27,28,29,30,31,32,40] the best coefficient of determination (*R*^2^) and the ratio of prediction to deviation (RPD) for assessing the SOM in moist soil (<35% *w*/*w* moisture) were about 0.86–0.88 [27] and 2.66 [29] respectively. Note that the assessments of SOM from relatively dry soil have achieved much better result with *R*^2^ and RPD of 0.892 ± 0.004 and 3.053 ± 0.056 respectively [15].

Apart from the correction approach as outlined in the previous paragraph, alternative methodology has been the discovery of the SOM related spectral bands [52,53,54,55,56,57,58,59] particularly those which are not greatly affected by the absorption feature of moisture in the soil [60,61,62]. There are numerous reports about the SOM-related absorption features in the vis-NIR spectral range and studies have revealed that spectral bands in the 800–1400 nm, 1600–1700 nm, 2100–2200 nm and 2300–2500 nm region [61] are relatively free of interference because of the presence of moisture content in the soil. However, a number of different sets of narrow wavebands, such as that centred at 1130 nm, 1350 nm, 1398 nm, 2210 nm [60], and more recent work reported that the set of 849 nm, 1681 nm, 2187 nm [58] and also another set at 917 nm, 991 nm, 1007 nm, 1996 nm, 2267 nm [59] gave good prediction of the SOM with *R*^2^ ~ 0.90. Recent study also expressed that new wavebands between 550–700 nm and 800–850 nm, 1700–1800 nm [62], narrow bands at 434 nm, 2368 nm and 2490 nm [55], and also those at 520 nm, 550 nm, 588 nm, 610 nm and 654 nm [54,56] were important for the estimation of SOM contents from the soil. The optimum SOM related bands were mostly obtained based on the one-dimensional (1-D) spectral correlation technique [52,54,56,59] and also through the optimisation methods using stepwise regression analysis in combination with various pre-processing- methods [55,56,58].

While there are numerous different sets of SOM-related bands reported in the literature that offers extremely useful information to workers in this field, it also introduces somewhat confusions to the users/researchers without knowing how to choose specific set of bands for their applications. This is particularly true when one deals with real world soil sample which may compose of numerous minerals and ingredients in the soil. This paper serves the sole purpose of introducing a simple, yet efficient method, to allow the researchers/users to visualise their data vividly and to allow crucial bands to be selected for their applications. The paper adopts the synchronous 2D correlation spectroscopy (2D-COS) method [63,64] from the spectroscopy research community, to help unveil the comparatively small spectral features of the SOM from the overwhelming moist content in the soil for the very first time. The goals of the paper have been set as follows: (i) A brief introduction of the working principles of the 2D-COS and to demonstrate how small features of difficult targets can be unveiled from huge background signals; (ii) to optimise the partial least square regression (PLSR) models by using a selected set of spectral bands obtained from the 2D-COS analysis for improving the robustness of the model prediction.

This paper is structured to highlight and to demonstrate the effectiveness of the 2D-COS technique for solving real-world problems. As such the present paper has employed a few arbitrarily selected moisture contents of soil samples to illustrate the working principles and methodology of the 2D-COS. The emphasis of the present work is to demonstrate the capability of the 2D-COS for unveiling subtle SOM features, and to allow their contents to be assessed more faithfully, despite of the presence of the overwhelmingly interfering spectral peaks from the moisture in the soil. The methodology can be applied for field trials when several batches of samples are collected locally from a field, the SOM contents of these samples can be deduced more faithfully through the few selected bands given by the 2D-COS analysis over these samples. The 2D-COS is a technique to extract the essence of the spectral band feature for further analysis.

## 2. Methods and Materials

### 2.1. The Synchronous Two-Dimensional Correlation Spectroscopy (2D-COS)

The basis of the two-dimensional correlation spectroscopy (2D-COS) is the formulation of a 2D matrix of the band-to-band covariance of all spectral vectors in the data set [63,64,65]. The method is originally designed for sensing the interaction between intermolecular functional groups and molecules, and the sensed information can be outputted structurally with much sharper and better resolved peaks, than the corresponding 1-D correlation [65]. The generalised 2D-COS method enables one to use numerous different types of external perturbations or physical stimuli that may induce perturbation/variations in the electro-optical spectrum. This set of dynamically perturbed spectra is then transformed into a set of 2D-COS by cross-correlation analysis. Since the 2D-COS analysis is historically evolved from statistical time-series analysis, a dynamic spectrum is retained for the description of the perturbation-induced spectral changes, even when the temporal aspect of the measurement is no longer relevant. Perturbations can be due to the effects of temperature, concentration, pressure or a time-dependent progression of spectral variations caused by the application of a stimulus. In the present case both the soil moisture and the SOM can be considered as perturbation variables in the spectral properties of the soil in this work. Consequently, the 2D-COS can be readily applied to analyse the spectral information due to the influences by the effects of moisture and SOM in the soil.

The external perturbation(s) *p* represents a measure of physical disturbances such as temperature, concentration, pressure, or moisture, etc. The spectral variable *v* can be any spectroscopic quantity such as wavelength in the infrared or NIR spectral range [66,67,68], Raman shift [69] or Terahertz shift [65,70], etc. In this paper the moisture and the SOM content have been treated as perturbation variables while the wavelength in the vis-NIR spectral region is utilised as the spectral variable of the soil/SOM mixture. When external perturbations are applied to the system, the stimulation due to the perturbation and the subsequent relaxation processes can be monitored through the electro-optical probe to obtain dynamic spectra until the system reaches equilibrium.

The dynamic spectrum $\tilde{y}$(*ν*, *p*) of the system under perturbation can be formally defined as:

(1) $$\tilde{y}\left(\nu ,p\right)=\{\begin{array}{c} y\left(\nu ,p\right)-\bar{y}\left(\nu \right)\;for\;p_{min}\leq p\leq p_{max}\;\\ 0\;otherwise \end{array}$$

where y(ν,p) is the perturbation-induced change of spectroscopic properties, such as the spectral absorbance. The variable *p* is bound within *p_min_* and *p_max_* and $\bar{y}\left(\nu \right)$ is the reference spectrum which can be set to be the averaged spectrum of the dynamic system:

(2) $$\bar{y}\left(v\right)=\frac{1}{p_{max}-p_{min}}\int_{p_{min}}^{p_{max}}y\left(v,\;p\right)dp$$

The fluctuation of the spectral properties between the perturbations (*p_min_*, *p_max_*) is then transformed into 2D-COS by using the correlation analysis method [63,64]. The perturbation due to moisture and SOM in the system modifies the spectroscopic properties of soil such as the variations of reflectance/absorbance (i.e., the change of the intensity of spectra), the shift of absorbance peak positions and their shapes. The 2D correlation spectrum X represents a quantitative comparison of two different spectral variables *ν*_1_ and *ν*_2_ over finite observation interval p between *p_min_* and *p_max_*:

(3) $$X\left(v_{1},v_{2}\right)=\tilde{y}\left(v_{1},p\right)\cdot \tilde{y}\left(v_{2},p^{\prime }\right)$$

where the < **^.^** > represents the covariance operator. Thus, the synchronous 2D correlation intensity $X\left(v_{1},v_{2}\right)$ gives an overall similarity between two separate spectral intensity variations $\tilde{y}\left(v,p\right)$ measured at two different spectral variables, *ν*_1_ and *ν*_2_, as the value of the perturbation *p* is changed.

**Figure 1:**
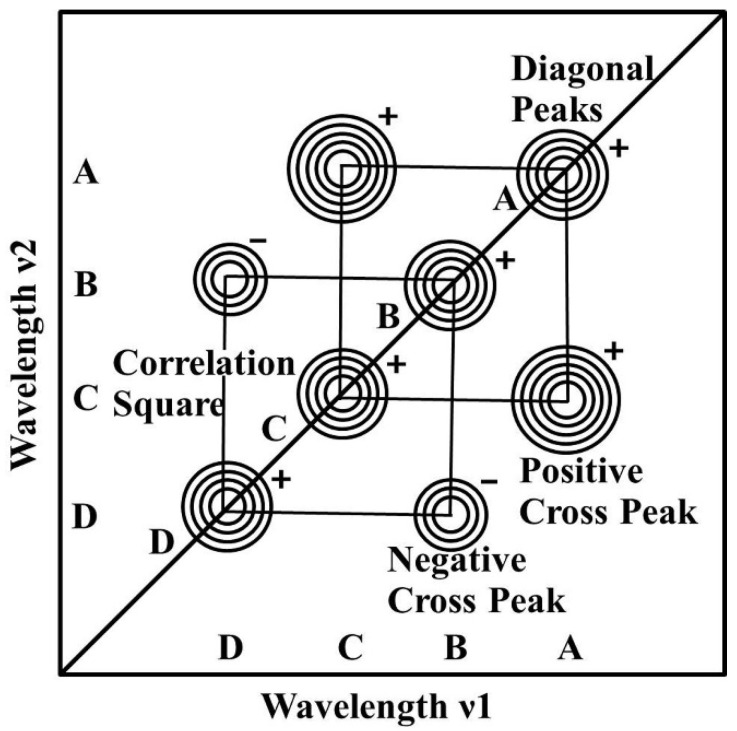
Outline the functionality and symbolic presentation of typical end results of the synchronous two-dimensional correlation spectroscopy (2D-COS).

A schematic example of the synchronous 2D correlation spectrum in the form of a contour map is shown in Figure 1. A synchronous spectrum is symmetric along the diagonal line corresponding to coordinates *ν*_1_ = *ν*_2_. Correlation peaks may appear at both the diagonal and off-diagonal positions. The peaks along the diagonal line are designated as auto-peaks. In the example spectrum shown in Figure 1, there are four distinct auto-peaks located at the spectral coordinates A, B, C and D. The magnitude of the auto-peak intensity, which is always positive, represents the overall extent of spectral intensity variation observed at the specific spectral variable *t* during the observation interval between *p_min_* and *p_max_*. Thus, any regions of a spectrum that changes the intensity under a given perturbation will show strong auto-peaks, while those remain nearly constant will develop few or no auto-peaks. While cross peaks are located at the off-diagonal positions represent simultaneous or coincidental changes of spectral intensities observed at two different spectral variables, *ν*_1_ and *ν*_2_. The signs of auto-peaks are always positive, but the signs of cross peaks can be either positive or negative. In the example shown both A, B, C and D form auto-peaks in the diagram. Both A and C, B and D form cross peaks. The sign of cross peaks at the spectral coordinates A and C is positive, indicating that both of bands increase (or decrease) in magnitude simultaneously. However, the cross peaks at the coordinates B and D is negative indicating that the intensity change of one component is increasing while the other is decreasing.

### 2.2. Soil Sample Preparation and Spectral Measurements

Total of 50 loam soil samples had been collected from the central of Shanxi Province (34°34′–40°44′ N, 110°14′–114°33′ S) in China and all samples were sampled from the top 5–15 cm of the soil surface for this study. The samples were first prepared by air-drying for a few days, then they were sieved through a 2.5-mm mess filter and were finally oven-dried at 106 °C for 6 h to eliminate the moisture in the soil samples. Subsequently all soil samples were dosed by five different moisture contents of approximately (oven dry, 5%, 10%, 15% and 17% *w*/*w*) according to the mass ratio:

(4) $$\theta_{m}=\frac{M_{w}}{M_{s}}\times 100$$

where $\theta_{m}$ is the mass moisture content (%); $M_{w}$ is the quantity of the water; $M_{s}$ is the quantity of the oven-dried soil samples. Because of the relatively small amount of water contents in the samples that have been employed in this study, fixed volume of distilled water was added to the pre-weighted soil sample drop by drop while the soil was continuously stirred to maximise the uniformity of the moisture across the entire batch of the sample. The mixture of the water and soil was then weighted again to deduce the water content of the mixture. After spectral requisition was completed, the water and soil mixture sample were dried in an oven at 106 °C for 12 h. The dried sample was then reweighted once more to confirm the consistency of the measured water content in each sample. The maximum moisture content in this work was set to 17% to avoid specular reflection due to the excess water on the surface of the soil. Previous study reported that the prediction of SOM was more reliable when the maximum soil moisture was less than 22% (*w*/*w*) in the loam soils [71].

Each of the 50 soil samples was divided into two equal parts: One was subject to destructive ground truth (GT) measurement for assessing the SOM content by using the conventional potassium dichromate method (NY/T 1121.6-2006 [https://www.chinesestandard.net/PDF/English.aspx/NYT1121.6-2006]); and the other part was designated for experiment. The GT measurement of the SOM contents was found ranging from 0.40% to 7.92%, with the mean value of 2.22% and standard deviation of 1.25%. The other parts of the soil samples were then dosed by various moisture concentrations.

The vis-NIR spectra of all 50 samples was obtained by the FieldSpec3 spectrometer (Malvern Panalytical Ltd., ASD Company, Las Vegas, NV, USA) within the spectral range of 350–2500 nm under the same experimental environment in the laboratory. The spectral resolution of the ASD was about 3 nm full-width-at-half-maxima (F WHM) at 700 nm, and 10 nm FWHM at 1400 nm and 2100 nm. Experimental condition consists of an external halogen light source illuminated at a few degrees off the normal plane of the samples. Each of the soil samples were spread out thinly (~5 mm thick) over the entire Petri dishes and three spectra were taken from one spot of the soil sample. The spectral acquisition was then repeated for another three different spots of the same soil sample. The mean of these nine spectra represented the vis-NIR spectrum of the sample. Each sample spectrum contains 2051 spectral bands, thus there are 50 × 2051 spectral features available for the characterisation of <50 discrete SOM contents over the entire sample size in this study. A splice correction had been applied for the correction of the step change of sensor response function at 1000 nm wavelength; and noisy data in the 350–400 nm and 2451–2450 nm wavebands were discarded prior to the data analysis.

### 2.3. Data Processing for Som Predictions and Assessment Indices

**Figure 2:**
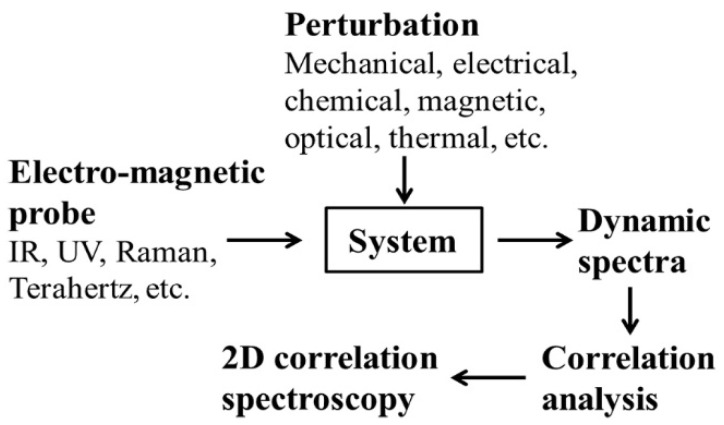
Schematic illustration of the flow chart for obtaining the 2D correlation spectra.

Note that the objective of this study is to assess the SOM content in the soil samples that contain high level of moistures which induce interference in the spectral analysis. Assessment of moisture level is not the goal of this work, the main emphasis of the current work is to choose the spectral features (i.e., the bands) that are associated with the SOM, but they are not greatly affected by the moisture spectral bands. To fulfil this aim, the proposed 2D-COS method is used. Once the most appropriate spectral bands have been established, the validity of these bands for SOM assessment is testified through the PLSR method. The SOM contents of all 50 samples were assessed using PLSR method. All samples were divided into five equal set and the SOM assessment was conducted under the five-fold cross-correlation scheme. The raw vis-NIR spectra is firstly analysed through the 2D-COS method as outlined in Section 2.1 using the 2Dshige version 1.3 software (2Dshige© Shigeaki Morita, Kwansei-Gakuin University, 2004–2005) [63,64,72], for the identification of the key wavelengths to be used for the establishment of the PLSR model. The experimental processing chain is schematically shown in Figure 2. The prediction model for the SOM detection is established through PLSR in the spectral range of 400–2450 nm using the MATLAB R2015a toolbox (The MathWorks, Inc., Natick, MA, USA). The PLSR utilises the SIMPLS algorithm [73] to establish the response and predictor loadings of the soil samples.

To demonstrate the effectiveness of the 2D-COS for enhancing the accuracy of the SOM content detection from moist soil, the experiment has been proceed under the following four schemes: (1) all spectral bands of the moist soil sample between 400–2450 nm have been utilised for building the PLSR model; (2) all spectral bands except the moisture bands have been employed; (3) all spectral bands except the moisture and weak noisy bands; and (4) just the SOM related bands (500–697 nm, 1546–1746 nm, 2100–2159 nm) are used as the input of the PLSR model.

The goodness of the prediction was assessed by comparing the predicted result with respect to the ground truth (GT) data via statistical indices:

Coefficient of determination (*R*^2^):

(5) $$R^{2}=1-\frac{\sum_{i=1}^{N}{\left(\hat{y_{i}}-y_{i}\right)}^{2}}{\sum_{i=1}^{N}{\left(y_{i}-\bar{y}\right)}^{2}}$$

where $\hat{y_{i}}$ is the predicted value, $y_{i}$ is the measured value; $\bar{y}$ is the mean of the all the $y_{i}$; *N* is the number of prediction data. The *R*^2^ measures how tight are the data points to the regression line, i.e., it gives the overall goodness of the regression model. However, a model depends on how reliable the calibration data is, hence there are three variants of *R*^2^: the coefficient of determination for the calibration (*R*_c_^2^), the coefficient of determination for the cross validation (*R*_c__v_^2^) and the coefficient of determination for the prediction/verification (*R*_p_^2^). The *R*_c_^2^ utilises the calibration data for the analysis and it measures the goodness (or the integrity) of the calibration data for the development of the regression model, while the *R*_p_^2^ shows the goodness of the verification (test) data. Hence the goodness of the *R*_c_^2^ has direct impact on the goodness of the *R*_p_^2^. Other assessment metrics such as the root mean square error of calibration (RMSEC), the root mean square error of cross validation (RMSECV), the root mean square error of prediction (RMSEP) and the ratio of prediction to deviation (RPD) are defined as:

(6) $$\;RMSEC=\sqrt{\frac{1}{I_{c}-f-1}\overset{I_{c}}{\underset{i=1}{\sum }}{\left(\hat{y_{p_{i}}}-y_{i}\right)}^{2}}\;RMSECV=\sqrt{\frac{1}{I_{cv}}\overset{I_{cv}}{\underset{i=1}{\sum }}{\left(\hat{y_{v_{i}}}-y_{i}\right)}^{2}}\;RMSEP=\sqrt{\frac{1}{I_{p}}\overset{I_{p}}{\underset{i=1}{\sum }}{\left(\hat{y_{p_{i}}}-y_{i}\right)}^{2}}\;and\;RPD=SD/RMSEP$$

where $I_{c}$, $I_{p}$ and $I_{cv}$ are the numbers of calibration, prediction and validation; f is the principle numbers; $\hat{y_{p}}$ is the prediction value derived from the spectral data using the PLSR; $y_{i}$ is the measured value from the laboratory measurement (i.e., the ground truth); $\hat{y_{v}}$ is the predicted value by the regression using the validation data set; SD is the standard deviation of the reference data (i.e., the calibration data set). RMSEC gives the residuals of the calibration (i.e., calibration error) and it measures the goodness of fit between the data and the calibration model. RMSECV gives the residuals of the validation (i.e., validation error). RMSEP measures the prediction error with respect to the reference value (i.e., the ground truth measurement values).

In this study the goodness of the prediction models has been assessed through the following assessment indices: (a) determination coefficient of calibration (*R*_c_^2^), (b) root mean square error of calibration (RMSEC), (c) determination coefficient of cross validation (*R*_cv_^2^), (d) root mean square error of cross validation (RMSECV), (e) determination coefficient of prediction (*R*_p_^2^), (f) root mean square error of prediction (RMSEP) and (g) the ratio of prediction to deviation (RPD). Generally, models with high *R*_c_^2^, *R*_cv_^2^ and *R*_p_^2^, and also high RPD with low RMSEC, RMSECV and RMSEP are indicative of good models. Since the RMSEP measures the mean of the sum of squares due to errors, i.e., the mean of the squared residual of the regressions, the RPD and *R*_p_^2^ together give a much better indication about the goodness and robustness of the model. When the RPD < 1.4 it is generally regarded as unacceptable predictions and when 1.4 ≤ RPD < 1.8 the prediction is considered as fair. Good predictions are generally in the range of 1.8 ≤ RPD < 2.0, and higher values of RPD 2.0 ≤ RPD < 2.5 generally indicative of very good predictions. When exceptionally high RPD ≥ 2.5 is obtained this implies the model gives excellent predictions [27].

## 3. Results and Discussions

### 3.1. Spectroscopic Characteristics of Soil and Moisture in the vis-NIR Spectral Range

**Figure 3:**
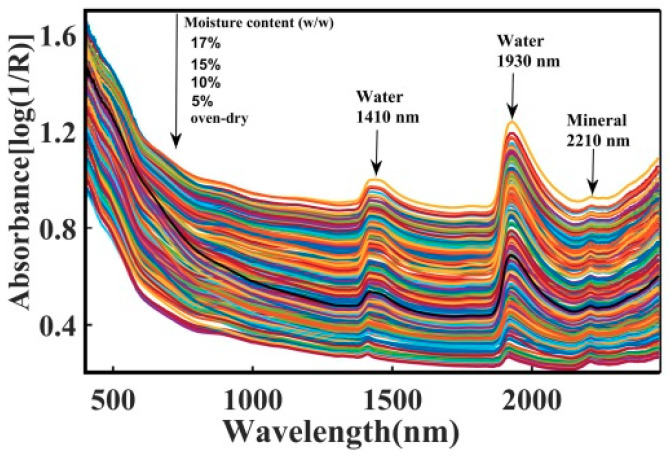
Shows the raw spectral (absorbance) plots of the soil samples that have been dosed by different levels of moisture.

To visualise how the moisture dominates the spectroscopic characteristics of moist soil, Figure 3 depicts the absorbance spectra of the oven-dried soil together with other samples dosed by moisture contents up to 17%. The overall absorbance of the soil increases with moisture content particularly in the near infrared region due to the increasing the quantity of the O-H functional groups [74]. It is seen that all spectra exhibit similar features with two prominent water absorbance peaks located at about 1410 nm and 1930 nm. The small feature at 2210 nm has been identified related to the minerals in the soil [57,75,76] while the two prominent humps at 1410 nm and 1930 nm are partly due to the absorptions by the vibrational of the hydroxyl group in the water molecules. The peak at the 1410 nm is the compound absorptions due to the soil mineral and organic matter [60,61,77]. The peak at around 2210 nm is seen insensitive to the increase of moisture content and it appears to be less correlated with the SOM content [36].

### 3.2. One-Dimensional Spectroscopy Analysis

**Figure 4:**
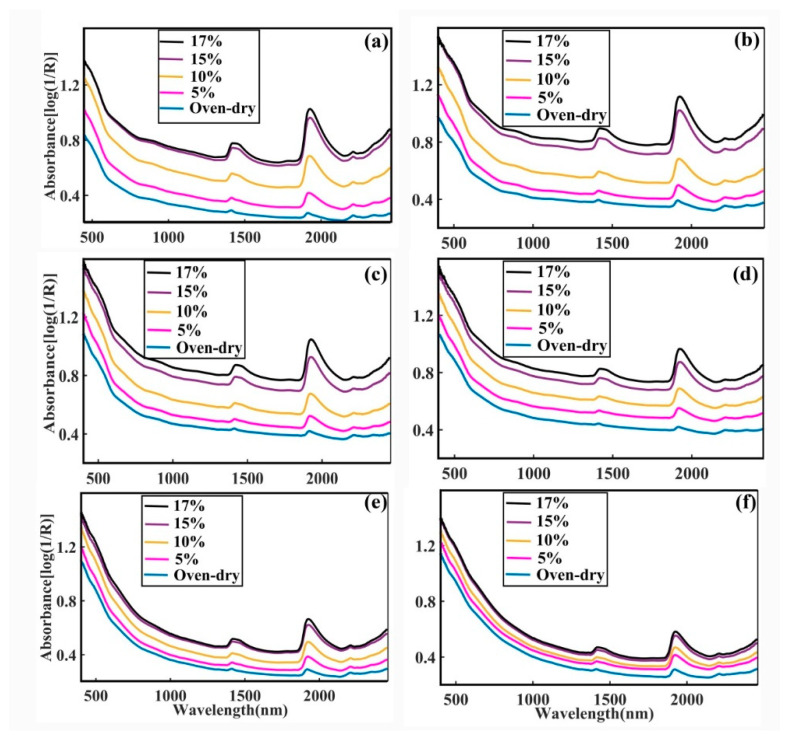
Shows the raw absorbance plots of soil samples that dosed by various moisture levels for the soil organic matter (SOM) content of: (**a**) 0.40%, (**b**) 1.12%, (**c**) 2.12%, (**d**) 3.35%, (**e**) 4.51% and (**f**) 7.92%.

Conventional spectroscopic analysis for SOM assessments routinely employs 1-D spectral data for building regression models [52,54,56,59] and they are often in conjunction with various pre-processing methods for performance enhancements [55,56,58]. To highlight the limitation of the 1-D spectroscopic analysis, Figure 4 presents the 1-D spectra of six soil samples with different SOM contents of (a) 0.40%, (b) 1.12%, (c) 2.12%, (d) 3.35%, (e) 4.51% and (f) 7.92% as function of various moisture contents (from oven-dry to 17% *w*/*w* moisture). All absorbance spectra in the figure exhibits very similar shape just like that shown in Figure 3, with two notable water bands near to 1930 nm and 1410 nm wavelengths [76]. The moist soil sample also shows an overall higher absorbance across the entire spectrum than that of the dry soil. This darkening (blackening) of the moist soil has been ascribed to the reduction of the reflection of light due to the sodium in the organic matter (humus), which disperses more readily and spreads over the soil particles when moisture exists in the soil [74]. When the moisture content is increased, the two peaks at the 1930 nm and 1410 nm are seen to red-shifted which is consistent with the increased absorptions by the increased amount of free water in the sample [36]. The small hump at around the 2210 nm wavelength is due to the minerals in the soil [36,57] and it is seen less affected by both the moisture and SOM contents. However, it is observed from this figure that the moisture absorption features in the vis-NIR region are broad and rather prominent in intensity. Furthermore, their spectral positions are so close to that of the SOM characteristic peaks (at about 1350–1400 nm [60]) that the prediction of the SOM content is expected to be severely interfered by the presence of these moisture features.

### 3.3. Two-Dimensional Correlation Spectroscopy Analysis

It is a technical challenge to analyse minute/subtle variations of features, particularly when they are convolved with other information due to various external perturbations. As mentioned previously, the synchronous 2D-COS technique is dedicated for unveiling subtle features due to external perturbations without the need of filtering or derivative operations. The main objective of this work is to demonstrate how the moisture, and the SOM, can be treated as perturbation variables within the context of correlation synchronous spectroscopy, and subsequently the SOM content can be revealed more accurately even when high level of moisture is present in the soil. Table 1 tabulates the details of the two types of perturbation variables that have been utilised for the analysis of the soil samples in this work. The list of the ‘variables’ in Table 1 is to illustrate the working principle of 2D-COS and how to choose the most appropriate bands for analysis. In this case six categories of samples have been randomly chosen from the 50 soil samples, and samples in each category are then randomly doped by different level of moistures to simulate various SOM contents in moist soil.

#### 3.3.1. Moisture-Induced Spectroscopic Features: Moisture as the Perturbation Variable

**Figure 5:**
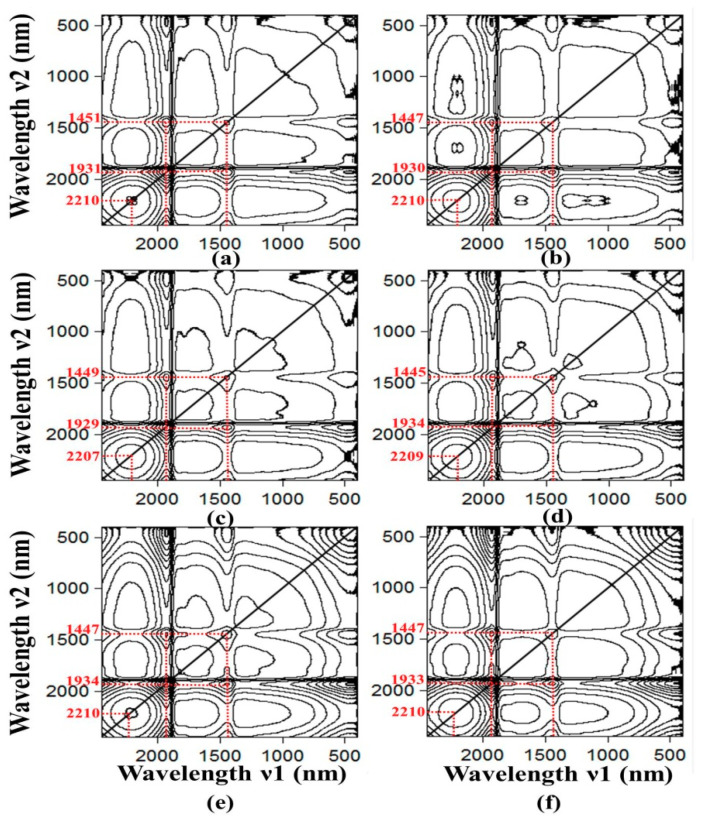
The contour plots of the synchronous two-dimensional correlation spectroscopy (2D-COS) of the soil samples that have SOM content of: (**a**) 0.40%, (**b**) 1.12%, (**c**) 2.12%, (**d**) 3.35%, (**e**) 4.51% and (**f**) 7.92%. Note that the 2D-COS is formed by using spectra of samples that have been dosed by various moisture contents in each category of SOM content.

**Figure 6:**
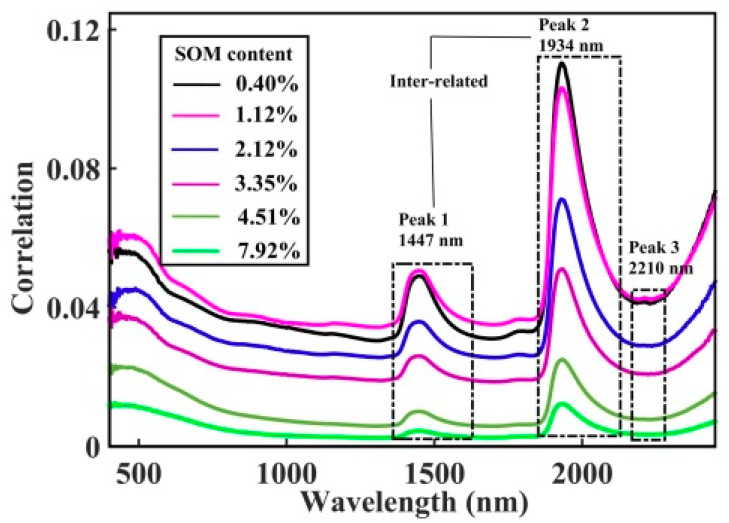
Plots the correlation strength along the diagonal lines of the synchronous two-dimensional correlation spectroscopy (2D-COS) in Figure 5 when moisture is treated as the perturbation variable in each set of the six different SOM content data.

First, it is necessary to establish the influence of the moisture on the spectroscopic property of the moist soil. Since we have two perturbation variables (i.e., the moisture and the SOM content) in this dataset, the analysis is proceeded with one variable to be kept constant; e.g., the SOM content is kept constant in order to study the effect of moisture as perturbation variable. The spectral data of all 50 samples has been regrouped into six sets, and each set contains a fixed amount of SOM content. Figure 5 depicts the 2D correlation synchronisation contour map of six sets of soil samples and each set contains samples with SOM contents of (a) 0.40%, (b) 1.12%, (c) 2.12%, (d) 3.35%, (e) 4.51% and (f) 7.92%. Each subplot is the 2D correlation contour map of samples that have been dosed by various moisture contents in each category of SOM content. Note that we are more interested in the local peaks and valleys of the covariance, hence the absolute magnitude of the contours in the figure have been omitted for clarity. In essence the covariance contour map depicts the correlated change due to the moisture, and the net effect can be seen more vividly by plotting the correlations, say, along the diagonal lines as shown in Figure 6 (through the matching peaks). The correlation strengths and the wavelengths of the peaks in Figure 6 are tabulated in Table 2.

According to the correlation intensities given by the contour plots, the influence of the auto-peak at around 1934 nm due to the moisture effect is seen to be the strongest. Second affected is the feature at around 1447 nm and the peak at 2210 nm is the least affected by moisture. It is known that the spectral features at 1934 nm originated from the interlayer water disturbances, while the one at 1447 nm is related to the hydroxyl groups and the peak at 2210 nm may be related to the O-H stretching vibrations and minerals. Figure 5 also reveals two cross-peaks at 1447 nm and 1934 nm which simultaneously increase their spectral intensities when the moisture content increases. This demonstrates that these two features are inter-related and are derived from the same origin, that is, due to the moisture in the soil.

As depicted in Figure 5, the moisture correlation strength of the auto-peaks is seen to reduce with the strongest correlation occurring at 1934 nm, then it becomes weaker at the 1447 nm and finally the weakest is found at the 2210 nm wavelength (see Table 2 for more information). At the same time, it is observed that the effect of moisture on the SOM content follows the same trend; when the SOM content is increased from 0.40% to 7.92%, the moisture correlation strength for these three peaks (1934 nm, 1447 nm and 2210 nm) are seen to reduce respectively from 0.1104 to 0.0122, from 0.0490 to 0.0044 and from 0.0417 to 0.0034, which equates to about 90% reduction of moisture effect over all three peaks when the SOM content is increased. These data give strong evidence that the soil moisture features overlap with the SOM characteristic peaks significantly, especially when the SOM content is in low concentration thus making the SOM content detection extremely difficult in these low SOM content situations (<1%). Consequently, based on this result it is suggested that the moisture bands at around 1934 nm, 1447 nm and 2210 nm should be removed to improve the accuracy of the SOM predictions.

#### 3.3.2. SOM-Induced Spectroscopic Features: SOM Content as the Perturbation Variable

**Figure 7:**
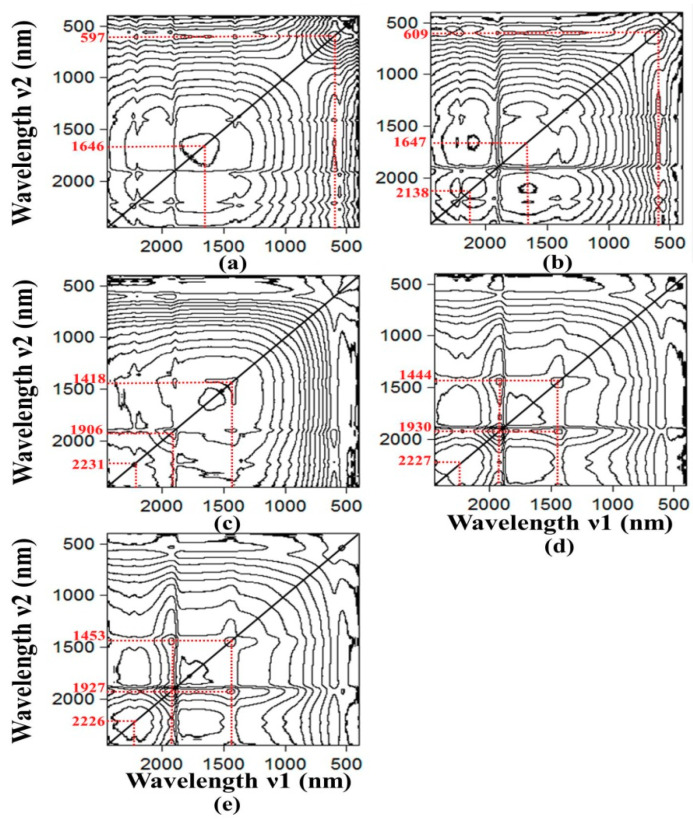
The contour plots of the 2D-COS of the soil samples with various SOM content (i.e., SOM as perturbation variable) for the moisture content of: (**a**) oven-dry, (**b**) 5%, (**c**) 10%, (**d**) 15% and (**e**) 17%. Note that the 2D-COS is formed by using spectra of samples that have various SOM content in each category of the moist soil sample.

**Figure 8:**
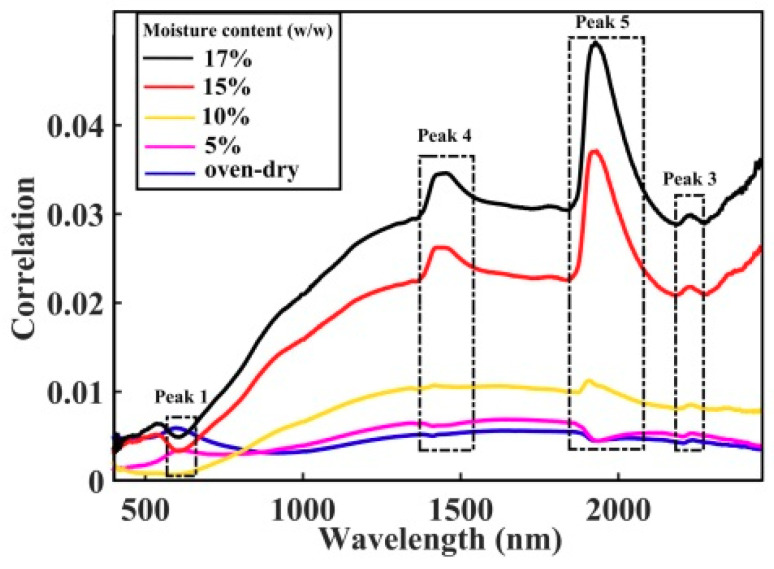
Plots the correlation strength along the diagonal lines of the 2D-COS in Figure 7 as function of the spectral wavelength, when the SOM content is treated as the perturbation variable in each set of the five different moisture content data. This correlation plot shows that the three bands at 597 nm, 1646 nm and 2138 nm are closely related to the SOM characteristic features.

Once the effects of perturbation due to moisture in the moist soil are understood, it is intuitive to investigate the influence of the SOM content on the spectral characteristics of moist soil. Similar to the last section, the spectral of all soil samples have been grouped into five sets and each has moisture content of (a) oven-dry, (b) 5%, (c) 10%, (d) 15% and (e) 17%. In this case we consider the SOM content in the soil as the perturbation parameter here. Figure 7 depicts the 2D-COS contour map of these five sets of data. Each contour map represents spectral correlations of the samples which contain various amount of SOM contents but all of them has the same moisture amount in each set of data. Figure 8 plots the correlation strength along the diagonal through the matching peaks, and the correlation strength of these auto-peaks are tabulated in Table 3.

Two distinct auto-peaks at around 597 nm and 1646 nm, have been seen clearly from the contour plot of the correlation synchronisation map of the oven-dried soil samples (see Figure 7a). A weak, but discernible peak begins to emerge at around 2138 nm when the moisture content is increased from oven-dry to 5% (see Figure 7b). This auto-peak is then seen red-shifted to about 2220 nm and at the same time, the correlation strength of this peak is increased from 0.0054 to 0.0299 (i.e., 450% increase) when the moisture content is further increased from 5% to 17% (see Figure 7b–e and Table 3). Two other auto-peaks near to 1934 nm and 1447 nm are also seen increasing in intensity as well as their correlation strengths, when the moisture content is further increased from 10% to 17% (see Figure 7c–e). This result may give further support of the analysis presented in the last section, that the three auto-peaks near to 1447 nm, 1934 nm and 2210 nm are greatly affected by the moisture in the soil. Previous study also suggested that the auto-peak at around 2138 nm might be related to the Al-OH and Mg-OH absorption bands [78]. Hence, it can be concluded from Figure 7; Figure 8 that the auto-peaks near to 597 nm, 1646 nm and 2138 nm are related to the SOM characteristic features. These SOM features are very weak in the vis-NIR spectral range and they could hardly be observed directly from the one-dimensional spectrum (please refer to Figure 3). However, it is shown in this study that this subtle SOM-related features can be enhanced, and subsequently revealed, by using the 2D-COS technique.

It is also observed that these SOM-related peaks seem to disappear when the moisture content is increased from 10% to 17% (see Figure 7c–e). As mentioned in the last paragraph, the SOM-related peak at 2138 nm can be seen at low moisture content of 5%, and this broad peak is seen to quench when the moisture contents increases to 10% (see Table 3 and Figure 8). At the same time three new peaks have been seen emerging at about 2230 nm, 1418 nm and 1906 nm. The latter two peaks are seen to red-shift by 30 nm as the moisture content is further increased to 17% (see Table 3). According to the analysis presented in the last section, these three peaks have been prescribed as related to the O-H bands of the moisture and their spectroscopic positions are strongly dependent on the degree of hydrogen bond formation. In other words, the red-shift of these peaks to longer wavelength is indicative of increasing bound water in the sample. Hence this result indicates that effect of moisture on the prediction of SOM is significant when the moisture content is higher than 15%. This result echoes the analysis present in the last section that the water moisture in the soil imposes significant interference for the prediction of SOM, particularly when the vis-NIR information is utilised for the analysis.

### 3.4. SOM Prediction by PLSR Method

The 2D correlation synchronisation analysis presented in previous sections have shown that, the three soil moisture characteristic bands at around 1934 nm, 1447 nm and 2210 nm wavelengths have imposed significant interference to the SOM features which are predominately located at approximately 597 nm, 1646 nm and 2138 nm wavelengths. The moisture features have induced strong spectroscopic distortions thereby affecting the accuracy of the SOM prediction severely. As mentioned in Section 2.3 (‘Data processing for SOM predictions and assessment indices’ section), the effectiveness of the SOM prediction is critically assessed here by using a selections of spectral bands as inputs for the PLSR model: (A) Base line situation: the use of all spectral bands from 400–2450 nm; (B) moisture band elimination: the use of SOM bands without the moisture bands that have been identified in the previous section (see Section 3.3.1 ‘Moisture induced spectroscopic features: moisture as the perturbation variable’); (C) the use of SOM bands without moisture and noisy bands from 400–500 nm and (D) SOM bands only: the employment of the SOM related bands that have been identified in the previous Section 3.3.2 (‘SOM induced spectroscopic features: SOM content as the perturbation variable’). Note that the four experimental configurations (A–D) are designed to illustrate the effect of the SOM prediction accuracies when different combinations of spectral bands have been used for the analysis. Specifically in experiment (D), where only the SOM featured bands have been selected like that of the traditional method commonly adopted by workers in the field [52,53,54,55,56,57,58,59,60,61,62], the experiment is designed to compare the impacts when SOM only bands are utilised with respect to the proposed band selection method as set out in Experiment (C).

Note that the Exp (A)–(D) use all 50 samples under the five-fold cross validation configuration, in which some of the samples have been randomly chosen for calibration of the PLSR model, and the other samples for validation. Since all 50 samples have been ground trothed by destructive methods (see Section 2.2 for more details), the goodness of the validation can be assessed. Note that the samples that have been utilised by Exp (A)–(D) are the same, but only the spectral bands that have been used for building the PLSR model are different. It is to reiterate that the samples chosen from the 6 different category of SOM levels have only been used for the selection of appropriate bands through the 2D-COS analysis and they are not the only sample that have been used for the calibration or prediction processing!

The analysis is performed using PLSR as described in Section 2.3 ‘Data processing for SOM predictions and assessment indices’ and the validation statistics of the four sets of experiments are shown in Table 4. It is found that the prediction of the SOM from the oven-dried soil samples is good when the SOM bands are used for building the PLSR model. However, the prediction is not good when moisture is present in the soil, and the RPD in Exp (D) is seen to be the ‘lowest’ when the SOM bands are used for the PLSR analysis (see Table 4). The very low PLSR statistics in this case is speculated mainly due to the large errors in the calibration with *R*_c_^2^ = 0.84, which is the lowest among the four experiments performed in this study. This is likely caused by the strong moisture absorption peak at the 2227 nm which subsequently dominates the SOM peak at 2138 nm. Furthermore, the two SOM peaks at about 600 nm and 1646 nm are seen to quench by the moisture absorptions when the water content in the soil is above 5% (see Table 3). The compound effect of these two occurrences corrupts the signal to noise ratio (SNR) of the spectroscopic data particularly within the SOM characteristic bands region (i.e., 500–697 nm, 1546–1746 nm and 2100–2159 nm) when moisture is present in the soil. The low SNR of the input data manifests itself of inducing large standard deviations in the prediction error (e.g., RMSEP = 0.67 the highest among the four experiments) and consequently having the lowest RPD = 2.34 (see Table 4) score in this study.

The very poor PLSR result by using only the SOM bands to build the model in Exp (D) may suggest that the moisture bands should be removed for the analysis. The removal of three moisture bands that have been found as according to the 2D–COS analysis in Section 3.3.1, i.e., the elimination of 1396–1498 nm, 1883–1985 nm and 2159–2261 nm bands as performed in Exp (B), gives almost 10% better *R*_c_^2^ than that of using only SOM bands (Exp. D) for building the PLSR model. The much improved *R*_c_^2^ in Exp (B) reduces the RMSEP by 30% (i.e., reduces RMSEP from 0.67 in Exp (D) to 0.47 in Exp (B)) which boosts the RPD by 24% (i.e., increase the RPD from 2.34 in Exp (D) to 2.89 in Exp (B)). The *R*_p_^2^ of Exp (B) is ~0.89 and with the RPD of 2.89, this result is compared very favourably with respect to previous studies which have achieved good *R*_p_^2^ of 0.86–0.88 [27,68] and RPD of 2.66 [66] by utilising model transfer methodology for the SOM assessment from moist soil. However, it is also seen from Table 4 that, the Exp (A) that utilises all bands ranging from 400–2500 nm data for building up the PLSR, has achieved seemingly even better result with RPD of 3.04 (i.e., ~5% better than that of Exp (B)). The better RPD in Exp (A) is believed to be due to the more robust calibration with *R*_c_^2^ of 0.93 (cf. the *R*_c_^2^ of 0.92 in Exp (B)) which helps to reduce the RMSEP by ~15% (i.e., reduces the RMSEP = 0.47 in Exp (B) to the RMSEP = 0.40 in Exp (A)). The reduction of the RMSEP in Exp (A) is seen as an instantaneously positive boost of the RPD.

Recall the 2D-COS analysis in Section 3.3.2 that reveals that the three bands at 597 nm, 1646 nm and 2138 nm have been found closely related to the SOM characteristic features. Particular attention is the one at 597 nm which is close to, but not the same as previously reported: the bands between 550 and 700 nm [62] and narrow bands at 588 nm, 610 nm [54,56] have been identified as crucial features for the assessment of SOM from moist soil according to previous studies. This band at 597 nm is seen to be rather broad with half width of ~100 nm according to the 2D-COS plot shown in Figure 8. Because of the very low quantum efficiency of the imaging sensor between 400 and 500 nm region, this weak signal band between 400 and 500 nm should be removed. Exp (C) utilises all the bands as Exp (B) but without the weak signal bands between 400 and 500 nm. It is seen from Table 4 that the *R*_p_^2^ has been enhanced by ~3%, and the RMSEP is reduced by ~10% (i.e., reduces the RMSEP = 0.47 in Exp (B) to RMSEP = 0.42 in Exp (C)). The dramatic reduction of the RMSEP by ~10% upon removal of noisy bands between 400 and 500 nm may suggest that the SOM band at about 597 nm is rather important for assessing the SOM content from moist soil. The RPD in Exp (C) is seen to increase by also ~10%, presumably it is the consequence of the large reduction in RMSEP, giving the best RPD of 3.19 over the four experiments! From the literature it is seen that the best RPD and the *R*_p_^2^ reported for assessing SOM from moist soil have been RPD < 2.66 and *R*_p_^2^ < 0.9 [27,66,68,78]. The very high RPD of 3.19 achieved here by using 2D-COS analysis without any pre-processing techniques may suggest that the 2D-COS is a powerful yet simple technique for the analysis of complex system like the assessment of SOM or minerals from cultivated soil. It is also noted that the result of the Exp(C) that uses 1648 spectral band (features) of data has achieved higher accuracy (e.g., the RPD and *R*_p_^2^) of ~5% better than that using all 2051 spectral bands for assessing the SOM content as performed in the Exp(A).

As mentioned in the introduction section, the main purpose of this work is to introduce the concept and to illustrate the effectiveness of the 2D-COS for the analysis of complex system. Hence other techniques, such as pre-processing using filters and spectral derivatives have not been applied here. Because of the broad intrinsic absorption line width in the vis-NIR spectral region, which is further broadened by the optics and instrumental noise of the sensor, the assessment of small features like the SOM will be enhanced if the spectral resolution can be improved. Current effort in our lab is working towards the enhancement of the spectral resolution by using data processing technique. It is believed that the higher resolution data will help to enhance the prediction of the SOM in the moist soil significantly. In our next paper we would like to explore how 2D-COS can be further exploited in conjunction with spectral dimension reduction, wavelet denoising and spectral derivative for the assessment of SOM.

## 4. Conclusions

This paper is aimed at introducing a simple yet effective technique for the analysis of complex systems more accurately and effortlessly. The system chosen in this paper is the assessment of soil organic matter (SOM) from moist soil samples by using spectroscopic technique. It is well-known that there are a few water absorption peaks overlapping with the SOM characteristic features in the visible-near infrared (Vis-NIR) spectral range. This makes the assessment of SOM from moist soil using spectroscopic technique very difficult. Through a simple synchronous 2D correlation spectroscopy (2D-COS) method, the interplay between the perturbation variables within a complex system can be untangled through a sequential analysis of the system when it is subjected to one perturbation at a time. By using the assessment of the SOM from moist soil system as an illustrative example, the contents of the SOM and the moisture level have been treated as perturbations to the soil system. The effects of the moisture, and the addition of SOM into the soil, have been analysed independently. The analysis is performed on the calibration data set that has known information of moisture and SOM contents in the samples. As demonstrated in Section 3.3.1 and Section 3.3.2, it has been shown that when the moisture is considered as the perturbation variable, the most prominent effect on the prediction of SOM content is the interference of the absorbance feature at the 1934 nm wavelength, and less interference is found for the wavelength at 1447 nm. Subsequently, the very small absorbance features associated with the SOM located at the 597 nm, 1646 nm and 2138 nm have been revealed by the 2D-COS when the SOM content is considered as the perturbation variable (see Section 3.3.2 for details).

As soon as the characteristic spectroscopic features of the SOM and the water bands have been revealed (by 2D-COS), this information is then utilised to build partial least square regression (PLSR) models for the prediction of the SOM content. In the present study, the effectiveness of SOM assessment by using information given by 2D-COS is assessed through the PLSR under a five-fold cross validation scheme. Four PLSR models has been established by using different sets of spectral bands to build the model: (A) all spectral bands has been used, (B) all the SOM bands but without water absorption bands, (C) all SOM bands but without water bands and also discards noisy bands between 400 and 500 nm and (D) only the SOM feature bands are used. Note that all the SOM and water bands utilised here are those revealed by the 2D-COS analysis. The PLSR result has revealed the worst prediction of SOM with RPD of 2.34, when the SOM bands alone (i.e., in Exp (D)) have been used for building the PLSR model. However, the use of the SOM band without water bands as performed in Exp (B) gives almost 24% improvement of the RPD, i.e., increase the RPD = 2.34 from Exp (D) to RPD = 2.89 in Exp (B). This indicates that the water absorption peaks impose severe interference to the SOM features. Further enhancement of the SOM assessment has been found by discarding noisy bands adjacent to the SOM feature as demonstrated in Exp (C), which achieves the best RPD of 3.19, almost 10% better than that Exp (B). The *R*_p_^2^ of Exp (C) is 0.92, which is seen to be the best over the four experiments performed in this study. It seems that the RPD = 3.19 and *R*_p_^2^ = 0.92 may be one of the best results for assessing SOM from moist soil ever been reported in the open domain as far as the authors are aware. The present result may suggest that the 2D-COS technique is effective for the analysis of complex system like the prediction of SOM from moist soil.

Since this work is aimed at introducing the 2D-COS technique for the analysis of complex system, pre-processing techniques have not been applied in the present paper. In our forthcoming paper we would like to exploit the 2D-COS in conjunction with spectral dimension reduction (e.g., through PCA and band selection etc.), wavelet denoising and spectral derivative for enhancing the prediction accuracy of SOM.

## Figures and Tables

**Table 1:** Outlines the two different types of perturbations that have been utilised for applying the synchronous two-dimensional correlation spectroscopy (2D-COS) analysis in this work.

Perturbation Variable	V1	V2	V3	V4	V5	V6
Moisture content (% *w*/*w*)	17 ± 0.1	15 ± 0.1	10 ± 0.2	5 ± 0.3	Oven-dry	-
SOM content- (% *w*/*w*)	7.92 ± 0.20	4.51 ± 0.15	3.35 ± 0.10	2.12 ± 0.02	1.12 ± 0.01	0.40 ± 0.02

**Table 2:** Tabulates the correlation strengths of the three main auto-peaks for the soil samples that have been dosed with various moisture content (i.e., moisture as perturbation variable): waveband uncertainty = ± 5 nm.

Samples with Soil Organic Matter (%)	Correlation Strength of Auto-Peaks		
	Peak 1	Peak 2	Peak 3
0.40 (a)	0.0490 (1451 nm)	0.1104 (1931 nm)	0.0417 (2210 nm)
1.12 (b)	0.0506 (1447 nm)	0.1032 (1930 nm)	0.0428 (2210 nm)
2.12 (c)	0.0358 (1449 nm)	0.0711 (1929 nm)	0.0290 (2207 nm)
3.35 (d)	0.0259 (1445 nm)	0.0511 (1934 nm)	0.0209 (2209 nm)
4.51 (e)	0.0100 (1447 nm)	0.0249 (1934 nm)	0.0078 (2210 nm)
7.92 (f)	0.0044 (1447 nm)	0.0122 (1933 nm)	0.0034 (2210 nm)

**Table 3:** Tabulates the correlation strength of the five main auto-peaks for the soil samples with various soil organic matter (SOM) content (i.e., SOM content as perturbation variable): waveband uncertainty = ±5 nm.

Moisture Content (%)	Correlation Strength of Auto-Peaks				
	Peak 1	Peak 2	Peak 3	Peak 4	Peak 5
Oven-dry (a)	0.0059 (597 nm)	0.0056 (1646 nm)	-	-	-
5% (b)	0.0034 (609 nm)	0.0069 (1647 nm)	0.0054 (2138 nm)	-	-
10% (c)	-	-	0.0085 (2231 nm)	0.0107 (1418 nm)	0.0112 (1906 nm)
15% (d)	-	-	0.0218 (2227 nm)	0.0262 (1444 nm)	0.0371 (1930 nm)
17% (e)	-	-	0.0299 (2226 nm)	0.0346 (1453 nm)	0.0494 (1927 nm)

**Table 4:** Shows the goodness of the SOM content predictions through the partial least square regression (PLSR) analysis under four different sets of spectral inputs for building the PLSR model.

PLSR Analysis	Spectral Range (nm) for PLSR Analysis	No. of Bands	*R*_c_^2^	RMSEC (%)	*R*_cv_^2^	RMSECV (%)	*R*_p_^2^	RMSEP (%)	RPD
A. Base line: all bands	400–2450	2051	0.93	0.32	0.88	0.46	0.88	0.40	3.04
B. SOM band without moisture bands	400–1396 1498–1883 1985–2159 2261–2450	1748	0.92	0.36	0.88	0.45	0.89	0.47	2.89
C. SOM band without moisture and noisy bands between 400–500 nm	500–1396 1498–1883 1985–2159 2261–2450	1648	0.92	0.36	0.88	0.45	0.92	0.42	3.19
D. SOM bands only	500–697 1546–1746 2100–2159	459	0.84	0.49	0.83	0.53	0.89	0.67	2.34
